# Exercise Interventions for Metabolic Diseases: An Analysis of the Evolution of Aerobic Exercise Bibliometrics in the Field of Type 2 Diabetes Mellitus

**DOI:** 10.3390/healthcare13172087

**Published:** 2025-08-22

**Authors:** Yang Li, Amin Ullah, Shuhao Fang, Donglin Liu, Zhenwei Cui, Guangning Kou

**Affiliations:** 1 Zhengzhou Key Laboratory of Sport Nutrition and Health, Zhengzhou University Research Center for Sport Nutrition and Health, Zhengzhou University, Zhengzhou 450001, China; liyang17503825963@gs.zzu.edu.cn (Y.L.); fangshuhao@gs.zzu.edu.cn (S.F.); 2 Department of Nutrition and Food Hygiene, School of Public Health, Zhengzhou University, Zhengzhou 450001, China; amin-nutrition@gs.zzu.edu.cn; 3 Faculty of Finance, City University of Macau, Macau 999078, China; f22090106882@cityu.edu.mo

**Keywords:** aerobic exercise, type 2 diabetes mellitus, research trends, exercise intervention, bibliometric analysis

## Abstract

**Background**: Type 2 diabetes mellitus (T2DM) is a major global public health challenge. Aerobic exercise (AE) can be a key strategy for non-pharmacological intervention in T2DM through multi-targeted modulation of glucose and lipid metabolism, inhibition of chronic inflammation, and reduction of oxidative stress. This study aims to investigate the current status of AE intervention in T2DM research and analyze its future evolution. **Methods**: Using the R-based bibliometric software package and the Java-based visualization software CiteSpace and VOSviewer, we analyzed the literature and cited references related to AE intervention in T2DM research included in the Web of Science Core Collection (WOSCC) and China National Knowledge Infrastructure (CNKI) from 2014 to 2024. **Results**: This study included a total of 882 relevant literature sources (488 of which were indexed in WOSCC and 394 in CNKI). From the perspective of research trends, the number of literature sources on AE interventions for T2DM has shown fluctuating changes over time. In terms of research output, the United States, China, and Canada are at the forefront. It is worth noting that, although China has a relatively high number of published papers, there is still a significant gap in terms of the depth of international collaboration and the presentation of results in top-tier journals. Among researchers, Dai Xia (China) and Riddell MC (Canada) are the scholars with the highest number of published articles in this field. Keyword analysis indicates that mechanisms such as oxidative stress, insulin resistance, inflammatory responses, and glucose metabolism disorders remain core research hotspots. Time-series analysis reveals that the research paradigm in this field has evolved from single exercise methods to comprehensive exercise prescription studies, and multi-dimensional intervention studies combining exercise, diet, and pharmacological interventions are emerging as new research frontiers. **Conclusions**: This study uses bibliometric methods to visualize and analyze the progress of AE in T2DM intervention research from a broader perspective, providing a scientific overview and macro-level predictions for the research landscape in this field.

## 1. Introduction

Data released by the International Diabetes Federation (IDF) in 2021 shows that the global prevalence of diabetes among adults is approximately 10.5%. China’s diabetes prevalence rate (12.8%) is higher than that of the global average, accounting for over 141 million patients, posing a significant challenge to public health. Research on the pathogenesis of diabetes has shown [1,2,3] that diabetes is a syndrome characterized by abnormalities in glucose and lipid metabolism, with the core manifestations being insufficient insulin secretion and/or insulin resistance [4,5,6]. Key underlying mechanisms include reduced phosphorylation levels of insulin receptor substrates, weakened activity of the phosphoinositide 3-kinase/protein kinase B signaling pathway, and impaired function of glucose transporter 4 (GLUT4) [7,8,9]. Survey results indicate that type 2 diabetes mellitus (T2DM) accounts for 90–95% of all diabetes cases, with pathological features including the following: in the early stages of the disease, pancreatic β-cells maintain blood glucose homeostasis through compensatory proliferation and excessive secretion, resulting in normal fasting C-peptide levels but elevated insulin secretion and insulin ratios; simultaneously, visceral adipocytes excessively release inflammatory factors such as tumor necrosis factor-α (TNF-α) and interleukin-6 (IL-6) and exhibit abnormal adipokine secretion (specifically manifested as leptin resistance and adiponectin deficiency) [10,11]. These factors collectively interfere with normal transmission of insulin signaling in skeletal muscle and liver, thereby impairing glucose regulation [12,13,14,15,16]. Additionally, patients with T2DM often have multiple complications. Studies have shown that the incidence of cardiovascular disease, kidney disease, and retinopathy in such patients is two to four times higher than in the general population [17,18,19].

As a metabolic disorder, T2DM should be managed according to the principle of “exercise–nutrition–medication” synergistic intervention [20,21]. Evidence-based medicine has confirmed that regular aerobic exercise (AE) is a level II evidence recommendation for the treatment of T2DM in the diabetes prevention and management guidelines of the World Health Organization (WHO) and the International Diabetes Federation (IDF) [22,23,24]. From a mechanistic perspective, AE significantly enhances glycogen synthase activity and the transport efficiency of GLUT4, thereby improving skeletal muscle glucose uptake capacity, leading to a significant reduction in glycated hemoglobin (HbA1c) levels, helping patients maintain blood glucose within target ranges and effectively delaying diabetes progression [25,26]. In terms of preventing and controlling complications, AE can improve vascular endothelial function, regulate lipid metabolism disorders, and reduce chronic inflammatory responses, thereby lowering the risk of complications associated with T2DM [27,28,29].

Bibliometrics and visualization analysis serve as tools for quantitative research, enabling in-depth exploration and pattern extraction from large volumes of literature data [30]. They are beneficial for revealing the knowledge structure, evolutionary path of research topics, and future development potential of specific medical fields [31]. This study focuses on literature related to AE in T2DM intervention research, systematically collecting and organizing it. Through intuitive and clear visualization, it presents the development trajectory of research hotspots in this field and the collaboration networks and evolutionary trends of core author groups, as well as collaboration patterns and dynamic changes among research institutions. Additionally, by integrating existing research findings and trends, this study scientifically predicts potential directions for breakthrough progress in this field. This not only provides an objective and comprehensive overview of the current application of AE in T2DM interventions, but also offers targeted and forward-looking theoretical guidance for future related research efforts.

## 2. Materials and Methods

### 2.1. Data Sources

The literature search was mainly conducted using two major databases: the Web of Science Core Collection (WOSCC) and China National Knowledge Infrastructure (CNKI). These two databases were selected because they comprehensively cover journals and literature and provide detailed data information, thereby meeting the search requirements of this study. For the WOSCC database, the following search strategy was developed to systematically collect studies related to AE interventions for T2DM: (TS = (“aerobic exercise” AND type 2 diabetes*) OR TS = (“aerobic training” AND type 2 diabetes mellitus*) OR TS = (“oxygen sports” AND type 2 diabetes*)). The search keywords were carefully selected to balance inclusivity and specificity, ensuring coverage of relevant studies while minimizing the inclusion of irrelevant results. In the CNKI database, a consistent search logic was applied: (SU% = “aerobic exercise” OR SU% = “endurance training” OR SU% = “aerobic training” OR SU% = “exercise therapy”) AND (SU% = “type 2 diabetes” OR SU% = “type 2 diabetes” OR SU% = “T2DM”). The search timeframe was set from 1 January 2014 to 11 October 2024, to ensure the inclusion of all relevant literature within this period and to comprehensively capture the research trajectory of the field from its early development to the present. Ultimately, 614 articles were retrieved from WOSCC, and 646 articles were retrieved from CNKI.

### 2.2. Inclusion and Exclusion Criteria

This study limits the scope of analysis in the WOSCC database to original research papers in English (document type: articles); the analysis of the CNKI database only includes journal articles. To ensure the relevance of the research and the validity of the data, both database searches exclude book chapters, letters, conference papers, theses, proposals, policy reports, patents, and newspaper articles, as such documents lack in-depth analysis of the research question and accurate mapping of the conceptual network. Therefore, they were not included in the scope of the study.

### 2.3. Screening Process

**Figure 1:**
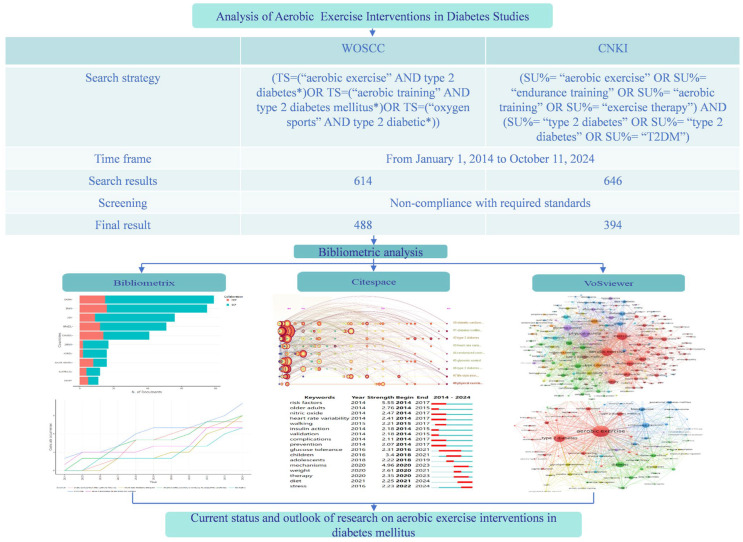
Literature screening and visualization process.

After completing the automatic screening process using database tools, the research team first conducted a manual review and cleanup of duplicate articles. Subsequently, based on pre-established inclusion and exclusion criteria, the articles were further evaluated. The specific screening process was as follows. Two evaluators independently reviewed the literature to determine its relevance to the research objectives. If there was a disagreement between the two evaluators, a third reviewer was introduced to participate in the assessment until a consensus was reached. Ultimately, only the literature that all evaluators unanimously agreed was consistent with the research theme was retained, resulting in the selection of 488 WOSCC articles and 394 CNKI articles. The screening process is illustrated in Figure 1.

### 2.4. Data Analysis and Visualization

This study used the R-based Bibliometric 4.4.2 package and the Java-based VOSviewer software (version 1.6.19) and CiteSpace software (version 6.3. R1) for qualitative data analysis and visualization. For ease of understanding, the analysis results from the CNKI database have been uniformly translated into English.

Using the Bibliometric software package based on the R language, we conducted a quantitative analysis of the publication output and citation impact of countries, journals, and authors. These indicators are commonly used in bibliometric research to assess scientific output (such as the number of published papers), impact (such as citation counts), and geographical and institutional distribution. Including these indicators helps provide a comprehensive overview of the research field. Network analysis using VOSviewer visualizes the collaboration networks among countries and authors in the field of AE intervention for T2DM research, clarifying their collaboration patterns. Keyword evolution analysis using CiteSpace aims to visualize current research hotspots and predict future trends [32].

## 3. Results

### 3.1. Trends in Publications and Core Journals

**Figure 2:**
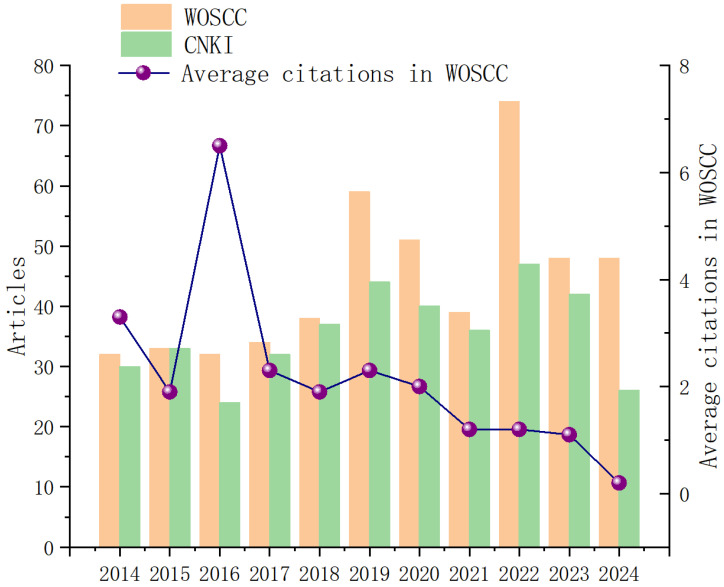
Trends in the number of publications and average citations from 2014 to 2024.

The number of papers published during a specific period can directly reflect the development trend of research in that field [33,34]. In the Web of Science Core Collection (WOSCC) database, there were 32 papers related to the topic of this study in 2014; from 2014 to 2024, the number of relevant papers in this database showed an overall fluctuating upward trend, with two distinct peaks in 2019 (59 articles) and 2022 (74 articles) (Figure 2). This characteristic indicates that research on aerobic exercise (AE) interventions for type 2 diabetes mellitus (T2DM) was extensively explored during the period from 2019 to 2022. The search results from the China National Knowledge Infrastructure (CNKI) database exhibit a similar evolutionary trend to those from WOSCC: the number of relevant papers published by Chinese scholars showed a fluctuating trend, with the highest number of papers published in 2022 (47 articles).

Meanwhile, the annual average citation count in the WOSCC database does not fully align with the trend in the number of published papers. While 2016 was the year with the highest citation count, it was also a year with a relatively low number of published papers. This may be because, as research progresses, the number of available references increases accordingly. Therefore, even in years with a lower number of published papers, the average citation count in the literature remains at a relatively high level [35].

**Figure 3:**
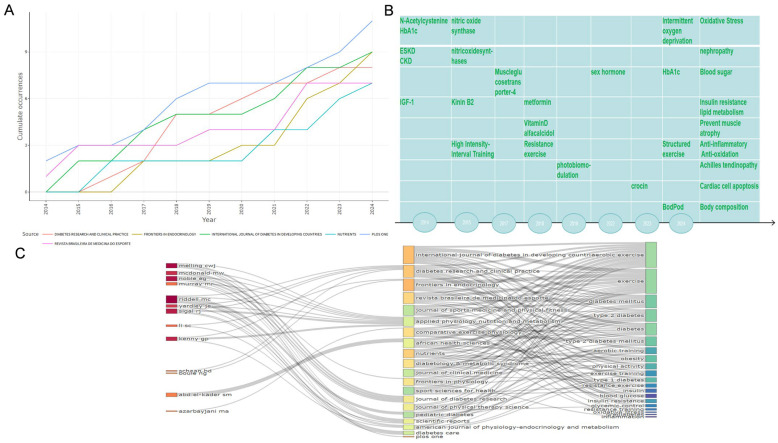
Top 10 journals and three field analyses. (**A**) Analysis of English literature source dynamics, (**B**) PLOS ONE publishing time and research content. (**C**) Authors, keywords, and English literature source network relationship.

All research articles on AE interventions in the T2DM field in the WOSCC database were published in 274 journals. Among the top ten core journals ranked by local citations (Table 1), DIABETES CARE had the highest total number of citations, reaching 1388; PLOS ONE published the most related articles (11), and the number of articles published in these core journals showed a year-on-year increasing trend. Further analysis of the 11 papers published in PLOS ONE (Figure 3A) revealed that 75% of the studies focused on three main themes: the inflammatory response, oxidative stress, and insulin resistance (Figure 3B). A three-dimensional mapping analysis conducted using Bibliometric software (Figure 3C) was used to construct a three-dimensional network composed of authors, journals, and keywords. Taking DIABETES CARE as an example, the papers this journal published mainly revolved around oxidative stress, lipid metabolism, and insulin resistance, while Professor Riddell MC, who published the most papers, also focused his research on these areas. The top 10 journals in terms of the number of related papers published in the CNKI database include the Chinese Journal of Tissue Engineering, The New World of Diabetes, and the Chinese Journal of Sports Medicine (Table 2).

### 3.2. Overview of Countries/Areas, Institutions, and Active Authors

**Figure 4:**
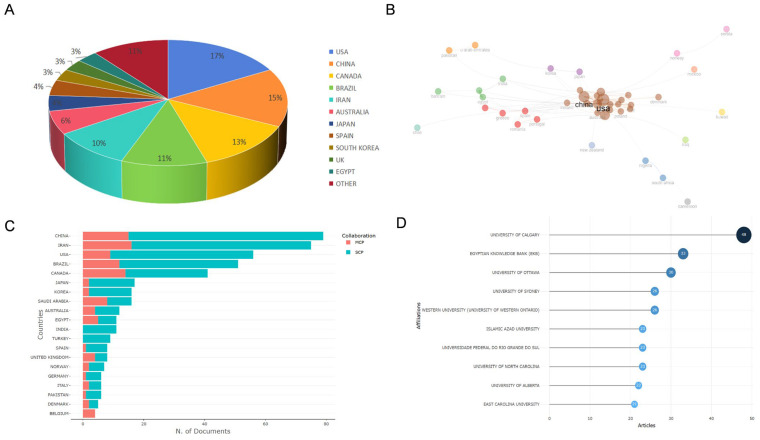
Visualization of analysis of countries and institutions. (**A**) Proportional distribution of global output. (**B**) Collaboration network of countries. (**C**) Countries of corresponding authors. (**D**) Articles from the top 10 institutions.

Research and analysis of high-output countries in a particular field can help objectively assess the disciplinary influence of various countries and related institutions, while also providing more reference material for subsequent academic exploration [36]. The analysis results from the Bibliometric software package show that the United States, China, and Canada rank first, second, and third, respectively, in terms of the number of papers published, with the total number of papers from these three countries accounting for 45% of the total number of papers in the field (Figure 4A). Among them, the United States published 269 papers, followed by China with 258 papers, with the total number of citations for papers from both countries exceeding 3000 (Table 3). The number of papers published is not completely positively correlated with the number of citations. For example, South Korea and Spain have similar numbers of papers published, but the number of citations for South Korean papers is almost four times that of Spain. This difference indicates that the influence of and attention to Spain’s relevant research results are still in their early stages, and there is significant space for improvement in terms of research precision, breadth, and depth. Further exploration is needed to enhance academic value and expand academic influence.

We also analyzed the cross-border collaboration network (Figure 4B) and the countries of affiliation of the corresponding authors (Figure 4C). Among these, multi-country collaboration papers (MCP) refers to the number of papers jointly authored by researchers from different countries, whereas single-country collaboration papers (SCP) refer to the number of papers jointly authored by researchers from the same country [37]. The analysis results show that academic collaboration in most countries is primarily domestic, with limited international exchange and collaboration, and MCPs account for less than one-third of all publications. Additionally, the data in Figure 4D indicates that the University of Calgary, the Egyptian Knowledge Bank (EKB), and the University of Ottawa account for nearly one-third of all publications.

The H-index is an important metric for evaluating a researcher’s academic achievements [38]. It refers to the number of papers published by a scholar that have been cited at least N times. Compared to simply considering the total impact factor (IF) or total number of citations, this metric provides a more comprehensive reflection of the quantity and quality of a researcher’s academic output. The top 10 authors ranked by paper output in the WOSCC database are listed in Table 4. These 10 scholars collectively contributed 73 papers, accounting for 20% of the total papers in the database. Among them, Professor Riddell MC from Canada topped the list with the highest number of papers. A review of Professor Riddell MC’s published works shows that his early research focused on the foundational aspects of exercise intervention for adolescent diabetes. In 2020, he systematically elucidated how metformin combined with AE improves glucose metabolism in T2DM patients by regulating molecular mechanisms such as insulin receptors and glucose transporter 4 (GLUT4) translocation, providing high-level evidence-based support for clinical practice.

**Figure 5:**
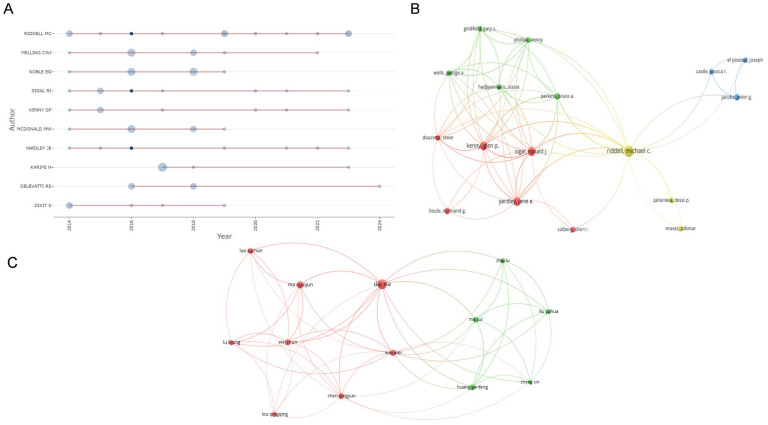
Visualization of active author analysis. (**A**) The timeline distribution of the top 10 most productive authors. (**B**) Cluster analysis of WOSCC authors’ cooperation. (**C**) Cluster analysis of CNKI authors’ cooperation.

Figure 5A shows the timeline distribution of the top 10 authors in terms of paper output and their collaboration networks, where node size corresponds to the number of papers published, and node color ranges from light blue to dark blue, representing citation counts from low to high, respectively. Within the scope of this study, Professor Riddell MC is an early pioneer in this field, with his related research spanning nearly 20 years. The collaboration network topology analysis, constructed using VOSviewer (Figure 5B,C) reveals that, in the WOSCC database collaboration network, 17 authors form a close collaboration, with Professor Riddell MC occupying the core hub position. The collaboration network in the CNKI database includes 13 authors, with Dai Xia and Wei Wei as core nodes. Focusing on the mechanism research of exercise therapy for T2DM, its development can be divided into two stages: 2000–2015 was the mechanism exploration stage, focusing on elucidating the three-dimensional association among oxidative stress, inflammatory factors, and protein expression in diabetic model rats after exercise intervention; and 2015–2023 was the multidisciplinary integration phase, which established a regulatory network system involving “exercise–gut microbiota–endothelial progenitor cells”.

### 3.3. Co-Citation Analysis of Highly Influential Literature and Research Hotspot Evolution

Conducting citation and co-citation analyses of the English-language literature [39,40], Local Citation Scores (LCSs) reflect the most frequently cited literature within the current research dataset, whereas Global Citation Scores (GCSs) highlight the characteristics of the most frequently cited literature in the WOSCC. When a piece of literature’s GCS value is high and its LCS value is relatively low, this often suggests that it may have a broader influence beyond its own research field.

Among the top 15 high-impact studies contributing most significantly to the dataset, 13 were published between 2014 and 2018, and 2 were published between 2019 and 2024 (Table 5). These studies primarily focused on the preventive and therapeutic roles of AE in the pathogenesis of T2DM, with a particular emphasis on key regulatory factors such as inflammatory cytokines (interleukin-6 (IL-6), tumor necrosis factor-α (TNF-α)), adipokines (adiponectin, leptin, anti-adiponectin), and nitric oxide (NO). Among these, a 2016 study by Sheri R. Colberg et al. (serving as the core basis for the American Diabetes Association [ADA] position statement) had the highest citation count (LCS = 64, GCS = 1509), with its high influence stemming from three key contributions. First, it systematically integrated clinical evidence on the effects of AE on glycemic control (glycated hemoglobin (HbA1c) reduction of 0.5–0.8%), lipid regulation, and improved vascular endothelial function in T2DM patients, providing core evidence-based support for the World Health Organization (WHO) and the International Diabetes Federation (IDF) to list exercise as a secondary intervention measure. Second, it proposed that “there is a dose-response relationship between exercise intensity and metabolic benefits,” correcting the traditional belief that “only low-intensity exercise is needed” and driving subsequent research to focus on new training models. Third, it established safety thresholds for exercise interventions (e.g., recommending blood glucose monitoring to avoid hypoglycemia risks), providing a practical framework for clinical practice. Following the publication of this literature, research on the “exercise intervention + clinical guidelines” theme increased by 189% between 2017 and 2024, directly shaping the research paradigm of “basic mechanisms → clinical translation” [41]. Additionally, a 2014 meta-analysis by Yasuaki Hayashino et al. (including 14 randomized controlled trials involving 824 patients) is also significant (LCS = 5, GCS = 167). This study systematically confirmed that exercise significantly reduces inflammatory markers (C-reactive protein, IL-6) in T2DM patients, clarified the effect of AE on reducing leptin levels, and provided core evidence for the mechanism that “exercise improves diabetes through anti-inflammatory pathways”. It also found that “longer exercise duration and higher frequency lead to more significant reductions in IL-6 levels,” revealing the association between intervention parameters and efficacy and driving research on optimizing exercise protocols. It was also observed that exercise has no significant effect on adiponectin and anti-adiponectin, thereby clarifying the scope of the study and providing direction for exploring alternative mechanisms [42], laying the foundation for research on the “exercise–inflammation–metabolism” association and profoundly influencing the exploration of mechanisms and clinical protocol design for exercise intervention in diabetes management. Notably, W. Mitranun et al.’s 2014 study [43] (GCS = 23), though cited less frequently, holds significant innovative value. This study compared the effects of continuous training (e.g., exercising at 70% of maximum heart rate for 40 min) and intermittent training (e.g., exercising at 85–90% of maximum heart rate for 2 min, followed by recovery at 50% intensity for 3 min, totaling 40 min, three times per week). It was the first randomized controlled trial to confirm that “interval training is superior to continuous training in improving microvascular reactivity (e.g., a 12.7% increase in NO synthesis) and blood glucose fluctuations,” challenging the traditional notion that “only prolonged moderate-intensity exercise is necessary” [43]. These findings directly prompted a surge in high-intensity interval training (HIIT)-related research after 2015 (accounting for 34.2% of total research over the past five years). They encouraged the academic community to focus on “personalized adaptation of exercise patterns” (e.g., considering age, comorbidities, and exercise type), marking a pivotal turning point in the transition from “single exercise patterns” to “precision prescriptions”.

### 3.4. Keyword Co-Occurrence Network and Clustering Evolution Analysis

**Figure 6:**
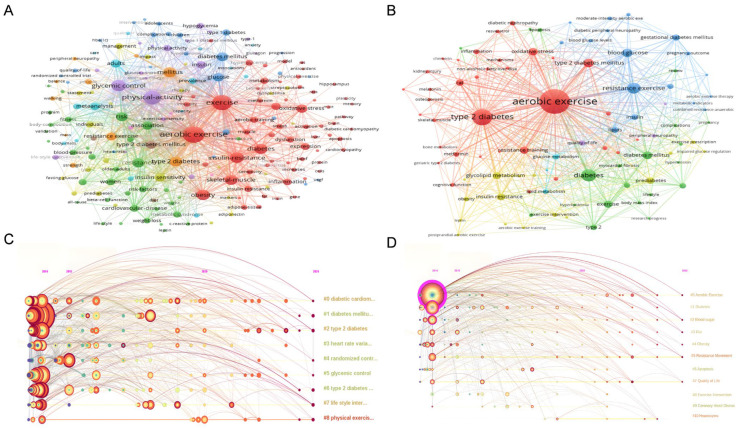
Keyword cluster analysis visualization diagram. (**A**) WOSCC keywords, (**B**) CNKI keywords, (**C**) WOSCC keyword cluster analysis timeline diagram, (**D**) CNKI keyword cluster analysis timeline diagram.

Keyword co-occurrence refers to the simultaneous appearance of two or more keywords in the same paper. This reflects the relationships and structure of these keywords within a specific research field, reveals research trends, and helps in identifying new disciplinary growth areas and trends [56]. Keyword clustering analysis was conducted using VOSviewer software. Each cluster consists of a circle and a colored-coded label indicating its affiliated cluster. In the WOSCC database (Figure 6A), “aerobic exercise,” “insulin resistance,” and “oxidative stress” form the core keywords. Among these, the blue and green clusters encompass terms such as “glucose homeostasis,” “insulin secretion,” and “metabolic syndrome,” which collectively embody core concepts related to the regulation of basal metabolic states in T2DM. The red cluster includes keywords such as “fat tissue insulin sensitivity,” “apoptosis,” and “inflammatory cascade reaction,” focusing on pathological mechanism studies related to exercise intervention, i.e., exploring physiological and biochemical abnormalities to reveal action pathways. Additionally, the presence of drug-related terms such as “metformin” in this cluster indicates that the academic community has started exploring combined exercise and drug intervention treatment strategies for T2DM [57,58]. The yellow cluster is represented by “exercise intensity threshold” and “physical activity patterns,” reflecting the empirical research dimensions of exercise intervention parameters. Notably, the appearance of terms such as “resistance training (RT)” and “HIIT” suggests that multi-dimensional exercise combinations may have positive application value in T2DM interventions [59,60,61]. In the CNKI database (Figure 6B), “aerobic exercise” appears most frequently, with 266 occurrences. The red cluster primarily focuses on the mechanisms by which AE improves T2DM and meta-analysis evidence; the blue, cyan, and yellow clusters involve topics such as “the antioxidant effects of resveratrol” and “diabetes,” reflecting research exploring the combination of exercise and dietary therapy for T2DM treatment [62,63]. The yellow cluster also includes exercise intervention parameters such as “resistance-endurance paired exercise” and “adaptive exercise patterns”; the purple cluster points toward clinical intervention practices, such as recommending a walking regimen of three times per week for 45 min each session for elderly patients or those with comorbidities to balance safety and compliance. For young, healthy individuals, HIIT three times a week for 20 min per session is more suitable for their busy lifestyles. These optimized intervention parameters provide robust literature support for the development of personalized exercise prescriptions [64,65,66,67,68].

Cluster analysis was performed on keywords from the WOSCC and CNKI using the log-likelihood ratio test algorithm, and a timeline diagram was plotted by selecting the “timeline” option [69]. The horizontal axis (x) of the timeline view represents the publication year, whereas the vertical axis (y) represents the cluster number. A more extended period indicates that the research field within that cluster emerged earlier and persisted for a longer duration. As shown in Figure 6C,D, the clusters “#1 Diabetes Classification,” “#2 Diabetes Complications,” and “#7 Lifestyle Intervention” in WOSCC have relatively more extended periods and are sustained research hotspots [70,71]. Meanwhile, CNKI data indicate that “#3 Glucose Dynamic Monitoring” and “#6 Inhibition of Pancreatic β-Cell Apoptosis” are major research hotspots for Chinese researchers [72,73]. Notably, research related to “#8 Gut Microbiota-Oxidative Stress Axis” has increased in recent years, indicating that this field is gradually shifting toward a multi-omics research paradigm [74,75,76].

**Figure 7:**
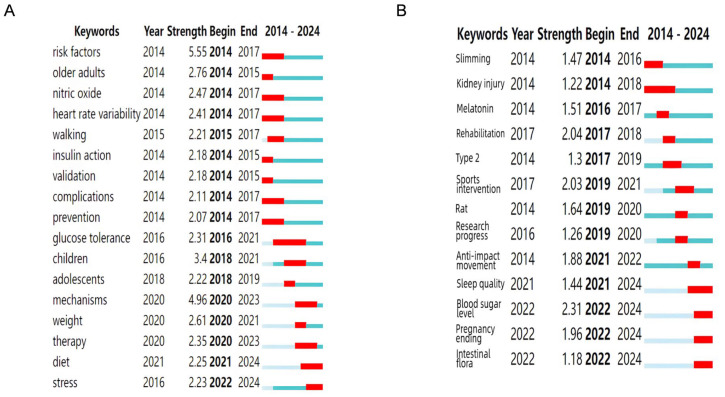
Keyword emergence. (**A**) The emergence of keywords in the WOSCC. (**B**) The emergence of keywords in CNKI.

Keyword prominence refers to a significant increase in the frequency of specific terms over a short period, indicating that the field has attracted high levels of attention from researchers during a particular period [77]. It reflects the cutting-edge developments and research trends in the field. The start and end times of the trend are marked as “start” and “end,” respectively, while “intensity” represents the strength of the keyword trend. The higher the intensity, the greater the impact [78]. Regarding keyword emergence, the light blue section represents the period of the included literature, while the red section indicates the start and end of a keyword’s emergence. In the WOSCC database (Figure 7A), the top five keywords sorted by prominence are “risk factors,” “elderly,” “nitric oxide,” “heart rate variability,” and “walking”. The research timeline shows that the literature from 2014 to 2018 primarily focused on the preventive value of exercise in the onset of T2DM and its mechanisms for delaying glucose metabolic abnormalities. After 2018, the research focus gradually shifted to an in-depth analysis of T2DM pathogenesis, precise identification of exercise intervention (AE) targets, and multimodal intervention strategies combining exercise with medication [79,80,81]. In the CNKI database (Figure 7B), the top five keywords by prominence were “weight loss,” “kidney damage,” “melatonin,” “rehabilitation,” and “type 2”. Between 2014 and 2022, a large number of studies focused on exploring endocrine dysfunction in adipose tissue, particularly the regulatory mechanisms of the leptin-adiponectin axis, as well as exercise-mediated prevention and treatment pathways for complications [82,83]. From 2022 to 2024, research shifted toward the regulatory mechanisms of the gut microbiota–oxidative stress pathway in type 2 diabetes and the role of the oxidative stress pathway in regulating type 2 diabetes. Research into the mechanisms of this pathway has yielded preliminary results, such as the pathway by which gut inflammation-induced microbiota metabolites regulate redox homeostasis through the nuclear factor-erythroid 2-related factor 2/antioxidant response element signaling pathway [84,85], which provides direction for targeted exercise intervention studies.

## 4. Discussion

This study is based on the Web of Science Core Collection (WOSCC) and China National Knowledge Infrastructure (CNKI) databases and conducts a quantitative analysis of research literature in the field of aerobic exercise (AE) intervention for type 2 diabetes mellitus (T2DM) from 2014 to 2024, systematically revealing significant patterns in this field in terms of research output, academic cooperation, and thematic evolution. These findings not only provide an objective overview of the development trends in this field, but also highlight the practical value of bibliometric methods in mapping scientific progress.

Our analysis revealed that the number of relevant articles in this field has been growing steadily over the past decade (the WOSCC database reached two peaks in 2019 and 2022, while the CNKI database peaked in 2022), a trend that aligns with the increasing global burden of T2DM. As mentioned in the introduction, the International Diabetes Federation (IDF) report indicates that the global prevalence of diabetes among adults reached 10.5% in 2021, with rates in China reaching as high as 12.8%. This severe public health crisis has driven sustained academic attention to non-pharmacological intervention methods (such as exercise therapy), becoming a key driver of increased research output. The peaks in literature volume in 2019 and 2022 may be related to the promotion of new clinical guidelines (such as the 2016 American Diabetes Association (ADA)’s position statement on exercise) and the impact of the COVID-19 pandemic, which further highlighted the importance of lifestyle interventions for chronic diseases, thereby increasing research attention in this domain.

From a geographical distribution perspective, analysis of national and institutional research output revealed the uneven nature of the global research landscape. China, the United States, and Canada lead in terms of the number of papers published, collectively contributing 40% of global research output, highlighting their dominant position in the field of metabolic disease research. It is worth noting that, despite China’s large number of published papers, there are still gaps in international collaboration (multi-country collaboration papers (MCP) rate below 30%) and publications in top-tier journals. These gaps may be related to differences in research content and focus. For example, WOSCC core journals emphasize basic mechanisms and interdisciplinary innovation, focusing on molecular pathways related to oxidative stress and insulin resistance; while the CNKI core journal literature emphasizes clinical translation and localized applications, with research primarily centered on validating the efficacy of interventions in specific populations (e.g., the hypoglycemic effects of AE in obese Chinese populations), optimizing traditional exercise protocols (e.g., the impact of Tai Chi on complications of T2DM), and integrating evidence from meta-analyses. Through analysis of author network collaboration clustering, it is evident that, in the WOSCC database, Professor Riddell MC and others serve as core nodes, with research focusing on molecular mechanisms (such as glucose transporter 4 (GLUT4) translocation and insulin signaling) [86]. In the CNKI database, collaborative research led by Dai Xia and Wei Wei focuses on the interaction between exercise and the gut microbiome [87], further highlighting the regional specificity of research clusters.

Through keyword and cluster evolution analysis, a clear and coherent developmental trajectory can be identified in this field. Core themes such as insulin resistance, oxidative stress, and inflammatory responses have consistently maintained high research interest, fully corroborating their critical roles in the metabolic abnormalities associated with T2DM. Since 2015, combined intervention protocols involving resistance training (RT) and high-intensity interval training (HIIT) have increasingly become a research focus. The most common combined training regimen involves three sessions per week, with all training content completed on the same day, divided into two parts: the first part consists of AE (such as cycling, swimming, or jogging) at an intensity of 60–70% of maximum heart rate for 30 min, and the second part consists of RT or HIIT, such as two sets of exercises targeting major muscle groups, with each set completed 8–12 times [88]. This exploration of diverse exercise prescriptions marks a significant shift in research paradigms, a trend validated by keyword co-occurrence analysis and citation analysis. Recent keyword timeline evolution analysis showed that, since 2022, there has been a significant increase in attention to the “gut microbiota–oxidative stress axis” and “metabolomics” in the CNKI literature, suggesting that future research may integrate multi-omics data (such as gut microbiota sequencing and serum metabolomics analysis) to customize personalized exercise intervention programs [89]. For example, by identifying microbial characteristics associated with exercise responsiveness, precise interventions can be achieved—combining AE with prebiotics targeting specific dysbiosis patterns to enhance metabolic improvement effects [90]. Notably, the emergence of keywords such as “drugs (e.g., metformin)” and “pharmaceutical food components (e.g., resveratrol)” [91] indicates that academic exploration of multi-dimensional intervention strategies is continuously expanding, providing robust evidence-based foundations for the development of clinical practice guidelines.

## 5. Limitations of the Study

This study explored the research prospects of AE intervention therapy in the treatment of T2DM through bibliometric analysis. However, the following limitations should be noted. First, this study only included literature from the WOSCC and CNKI databases. Although these two databases are widely used, they do not cover all relevant literature, potentially leading to an incomplete depiction of the current research landscape. Second, the selection of the time range may be biased. To track the latest research trends, the study limited the time frame to 2014–2024, which may have resulted in the exclusion of foundational studies from earlier periods that made significant contributions to the field. Third, the search strategy requires improvement. The search terms used in the study may not have covered all relevant expressions, inevitably leading to the exclusion of some relevant literature. Fourth, there are language and regional biases. This study is based solely on the English and Chinese literature, which may overlook important research findings from non-English and non-Chinese-speaking countries, thereby limiting the universality of the research results. Fifth, there are constraints on the analysis of exercise prescription variables. This study explored key prescription variables, such as exercise intensity, frequency, duration, and type, based on bibliometric data and content analysis of the key literature. However, the research methodology inherently relies on details provided in the original literature titles, abstracts, and keywords. Due to differences in reporting standards across different studies, this may limit the ability to systematically quantify and compare these parameters across the entire literature database. Sixth, citation frequency, H index, and other indicators are affected by the publishing strategy, citation habits, and journal influence. Although these indicators have important reference value, they should not be regarded as the only yardstick to measure the impact or quality of research. In addition, although bibliometric analysis can effectively capture research trends, it cannot independently verify causal relationships between variables. Therefore, the correlations and trends revealed by this method should be interpreted with caution.

## 6. Conclusions

This study employed bibliometric analysis methods to conduct a visualization analysis of research literature in the field of aerobic exercise (AE) intervention for type 2 diabetes mellitus (T2DM) from the Web of Science Core Collection (WOSCC) and China National Knowledge Infrastructure (CNKI) databases between 2014 and 2024, systematically revealing the development trends in this field. The findings indicate that the number of articles in the field of AE intervention for T2DM has shown a fluctuating growth trend over time. From the perspective of global research output patterns, China, the United States, and Canada account for 45% of the total number of published papers, with the United States leading in terms of citation influence. This suggests that China needs to further strengthen international cooperation, continuously improve research quality, and enhance its global academic influence and dominance in this field. Notably, the research paradigm in this field has undergone a significant shift in recent years, evolving from a single exercise regimen toward comprehensive exercise prescriptions and expanding to multi-dimensional strategies combining exercise, diet, and pharmacological interventions. The findings based on the bibliometric data clearly outline the development trajectory, key themes, and collaboration models within the field, providing a comprehensive overview with practical reference value for advancing T2DM exercise intervention research.

## Figures and Tables

**Table 1:** Top 10 journals cited by WOSCC.

Rank	Source	Local Citations	Articles	H-Index	Impact Factor (2024)
1	*DIABETES CARE*	1388	5	5	14.8
2	*MED SCI SPORT EXER*	540	4	4	4.1
3	*DIABETES*	495	2	5	6.2
4	*DIABETOLOGIA*	456	3	2	8.4
5	*J APPL PHYSIOL*	384	4	3	3.3
6	*PLOS ONE*	318	11	6	2.9
7	*CIRCULATION*	278	3	3	35.6
8	*JAMA-J AM MED ASSOC*	276	1	5	63.5
9	*DIABETES RES CLIN PR*	251	8	5	6.1
10	*J CLIN ENDOCR METAB*	239	4	2	5.0

**Table 2:** Top 10 journals in terms of the number of articles published in CNKI.

Rank	Periodicals	Volume of Publications
1	*Tissue Engineering Research in China*	13
2	*The New World of Diabetes*	11
3	*Chinese Journal of Sports Medicine*	11
4	*Chinese Journal of Gerontology*	10
5	*Chinese Journal of Applied Physiology*	10
6	*Chinese Journal of Rehabilitation Medicine*	9
7	*Bulletin of Scientific and Technical Literature on Sports*	8
8	*Contemporary Sports Technology*	7
9	*Sichuan Sports Science*	6
10	*Chinese and foreign medical research*	5

**Table 3:** Articles and total citations from the top 10 producing countries.

Rank	Countries	Articles	Total Citations
1	USA	269	2571
2	CHINA	258	817
3	CANADA	226	649
4	BRAZIL	202	608
5	IRAN	192	546
6	AUSTRALIA	84	141
7	JAPAN	65	313
8	SPAIN	58	47
9	SOUTH KOREA	47	206
10	UK	44	167

**Table 4:** Top 10 authors with the most published papers.

Rank	Author	Country	Articles	Total Citations	H-Index
1	RIDDELL MC	CANADA	12	85	9
2	MELLING CWJ	CANADA	8	14	6
3	NOBLE EG	CANADA	8	14	6
4	SIGAL RJ	CANADA	8	71	6
5	KENNY GP	CANADA	7	7	5
6	MCDONALD MW	USA	7	14	6
7	YARDLEY JE	CANADA	7	73	4
8	KARIMI H	USA	6	5	5
9	DELEVATTI RS	BRAZIL	5	8	4
10	DIXIT S	INDIA	5	14	5

**Table 5:** Top 15 cited English-language articles.

No.	Title	DOI	Year	LCS	GCS
1	Physical activity/exercise and diabetes: a position statement of the American Diabetes Association [41]	https://doi.org/10.2337/dc16-1728	2016	64	1509
2	Effects of exercise on C-reactive protein, inflammatory cytokine and adipokine in patients with type 2 diabetes: a meta-analysis of randomized controlled trials controlled trials [42]	https://doi.org/10.1016/j.metabol.2013.08.018	2014	5	167
3	Continuous vs. interval training on glycemic control and macro- and microvascular reactivity in type 2 diabetic patients [43]	https://doi.org/10.1111/sms.12112	2014	19	23
4	Exercise training and endothelial function in patients with type 2 diabetes: a meta-analysis [44]	https://doi.org/10.1186/s12933-018-0711-2	2018	1	98
5	Short-term high-intensity interval and moderate-intensity continuous training reduce leukocyte TLR4 in inactive adults at elevated risk of type 2 diabetes [45]	https://doi.org/10.1152/japplphysiol.00334.2015	2015	0	90
6	Effect of aerobic exercise intensity on glycemic control in type 2 diabetes: a meta-analysis of head-to-head randomized trials [46]	https://doi.org/10.1007/s00592-016-0870-0	2016	0	85
7	Effect of aerobic exercise on peripheral nerve functions of population with diabetic peripheral neuropathy in type 2 diabetes: a single blind, parallel group randomized controlled trial [47]	https://doi.org/10.1016/j.jdiacomp.2013.12.006	2013	9	84
8	Aerobic exercise training reduces arterial stiffness in metabolic syndrome [48]	https://doi.org/10.1152/japplphysiol.00151.2014	2014	1	83
9	Lag time remains with newer real-time continuous glucose monitoring technology during aerobic exercise in adults living with type 1 diabetes [49]	https://doi.org/10.1089/dia.2018.0364	2018	2	76
10	Exercise improves gait, reaction time and postural stability in older adults with type 2 diabetes and neuropathy [50]	https://doi.org/10.1016/j.jdiacomp.2014.04.007	2014	1	71
11	Effect of aerobic exercise and diet on liver fat in pre-diabetic patients with non-alcoholic-fatty-liver-disease: a randomized controlled trial [51]	https://doi.org/10.1038/s41598-017-16159-x	2017	1	69
12	Effects of high-intensity interval and moderate-intensity continuous aerobic exercise on diabetic obese patients with nonalcoholic fatty liver disease: a comparative randomized controlled trial [52]	https://doi.org/10.1097/MD.0000000000019471	2020	2	67
13	The effects of aerobic exercise training at two different intensities in obesity and type 2 diabetes: implications for oxidative stress, low-grade inflammation and nitric oxide production [53]	https://doi.org/10.1007/s00421-013-2769-6	2014	6	67
14	Exercise in children and adolescents with diabetes [54]	https://doi.org/10.1111/pedi.12176	2014	2	66
15	A randomized controlled trial on the effectiveness of 8-week high-intensity interval exercise on intrahepatic triglycerides, visceral lipids, and health-related quality of life in diabetic obese patients with nonalcoholic fatty liver disease [55]	https://doi.org/10.1097/MD.0000000000014918	2019	0	65

## Data Availability

More data will be provided on request to the corresponding author.

## Abbreviations

The following abbreviations are used in this manuscript:

T2DM	Type 2 Diabetes Mellitus
AE	Aerobic Exercise
WOSCC	Web of Science Core Collection
CNKI	China National Knowledge Infrastructure
GLUT4	Glucose Transporter 4
TNF-α	Tumor Necrosis Factor-α
IL-6	Interleukin-6
RT	Resistance Training
HIIT	High-intensity Interval Training
HbA1c	Glycated Hemoglobin
NO	Nitric Oxide
ADA	American Diabetes Association
IDF	International Diabetes Federation
WHO	World Health Organization
MCP	Multi-Country Collaboration Papers
SCP	Single-Country Collaboration Papers
EKB	Egyptian Knowledge Bank
IF	Impact Factor
